# Autoimmune Hepatitis: Tolerogenic Immunological State During Pregnancy and Immune Escape in Post-partum

**DOI:** 10.3389/fimmu.2020.591380

**Published:** 2020-09-24

**Authors:** Amber G. Bozward, Grace E. Wootton, Oskar Podstawka, Ye H. Oo

**Affiliations:** ^1^Centre for Liver and Gastroenterology Research, NIHR Birmingham Biomedical Research Centre, Institute of Immunology and Immunotherapy, University of Birmingham, Birmingham, United Kingdom; ^2^Centre for Rare Diseases, European Reference Network ERN Rare-Liver, Birmingham, United Kingdom; ^3^Liver Transplant and Hepatology Unit, University Hospitals Birmingham NHS Foundation Trust, Birmingham, United Kingdom

**Keywords:** pregnancy, tolerance, autoimmune hepatitis, placenta, memory T cells, regulatory T cells

## Abstract

The maternal immune system engages in a fine balancing act during pregnancy by simultaneously maintaining immune tolerance to the fetus and immune responses to protect against invading organisms. Pregnancy is an intricately orchestrated process where effector immune cells with fetal specificity are selectively silenced. This requires a sustained immune suppressive state not only by expansion of maternal Foxp3+ regulatory T cells (Tregs) but also by leaning the immune clock toward a Th2 dominant arm. The fetus, known as a semi-allograft or temporary-self, leads to remission of autoimmune hepatitis during pregnancy. However, this tolerogenic immune state reverts back to a Th1 dominant arm, resulting in post-partum flare of AIH. Various hormones play a significant role in endocrine-immune axis during pregnancy. The placenta functions as a barrier between the maternal immune system and the fetus also plays a pivotal role in creating a tolerogenic environment during pregnancy. We review the evidence of immune tolerance during pregnancy and immune escape at post-partum period, focusing on patients with autoimmune hepatitis.

## Autoimmune Hepatitis

Autoimmune hepatitis (AIH) is an immune mediated chronic liver disease with unknown etiology (1). Its prevalence ranges from 16 to 18 cases per 100,000 people in Europe (2). AIH is characterized by an aberrant innate and adaptive immune response targeting liver autoantigens toward liver parenchyma cells (hepatocytes) and epithelial cells (biliary epithelium) which perpetuate to liver inflammation. If left untreated, AIH leads to chronic hepatitis, resulting in progressive fibrosis and eventually leading to liver cirrhosis and cancer.

An individual’s susceptibility to AIH can be influenced by many factors including genetics and environmental surroundings. Clinical manifestations of AIH vary from asymptomatic to fulminant presentation (1–3). Genetic predisposition is thought to be governed by the HLA haplotype; this determines the formation of the antigen binding site of MHC class II which in turn presents autoantigens to CD4 T-cells (4, 5). Environmental triggers, such as Aryl hydrocarbon ligands, can shape the T-cell immune response to direct it at liver antigens, leading to inflammation and scarring downstream (6–8).

Autoimmune hepatitis predominantly affects females; a significant proportion of which are young women of child-bearing age (9, 10). Previously, pregnancy in AIH patients was rare as it was viewed as high risk due to concerns of reduced fertility and unknown safety profiles of immunosuppressive regimens used in AIH (11). However, pregnancy in patients with AIH is increasingly common due to recent advancements in *in vitro* fertilization, advanced and protocolized clinical management between the hepatologist and obstetrician, pre-pregnancy counseling and an in-depth understanding of drug safety profiles. Careful clinical monitoring of both mother and child before conception (pre-pregnancy counseling) as well as during pregnancy, with combined care from both liver and obstetric teams is crucial to achieve a good outcome, especially in cirrhotic patients. Post-partum follow up within 8 weeks is also prudent to detect and manage the flare up of AIH after delivery.

Pregnant AIH patients experience disease remission during pregnancy but often endure flare activity of AIH in the early post-partum period (9, 12, 13). However, the underlying immunological mechanism is still unresolved. In this review, we discuss published evidence in order to understand immune tolerance in pregnancy and immune escape in post-partum period in AIH patients.

## Hormone and Liver Enzyme Changes During Pregnancy

The immune system functions to protect the body from harmful foreign antigens. The innate and adaptive immune system work in harmony to carry out these protective processes.

Cells involved in the innate immune response include dendritic cells (DC’s), natural killer (NK) cells, neutrophils and macrophages. DC’s function as antigen-presenting cells (APC’s) and NK cells secrete cytokines and function as killer cells. The innate cells then crosstalk with T cells, cytotoxic CD8 T cells, helper CD4 T cells and antibody secreting B cells. Regulatory T-cells function to prevent attack of self-antigens.

There are different phases during pregnancy, each of which can be characterized by notable immunological shifts along with changes in hormonal levels (14). Progesterone (P4) is critical in the establishment and maintenance of pregnancy. Rising levels stimulate uterine homing of NK cells and up-regulation of HLA-G gene expression, which are the ligands for NK inhibitory receptors (15). It leads to the secretion of Th2 cytokines and inhibition of Th1 development which suppresses the maternal immune response to the fetus (16). In addition, P4 promotes fetal growth by CD8+ T cell modulation by placental heme oxygenase 1 (Hmox1) expression (17). Estrogen rises at a similar rate. The implantation of the blastocyst into the endometrial tissue creates a cycle of cell repair, death and regeneration thus creating an inflammatory environment to remove cellular debris (18, 19). Human chorionic gonadotrophin (hCG) peaks between weeks 8 and 10 and then plateau’s as pregnancy progresses (20, 21).

**FIGURE 1:**
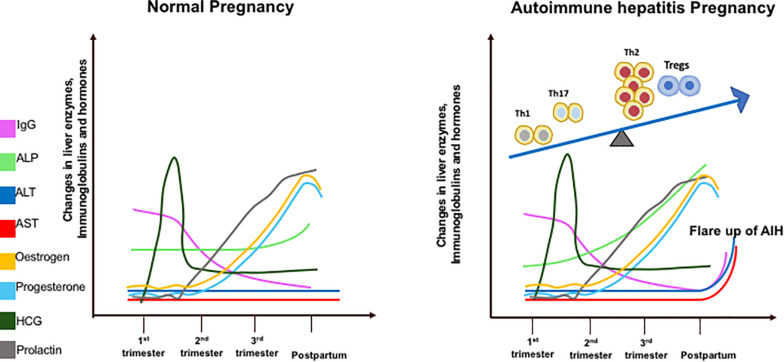
Diagrammatic illustration of liver enzymes (aspartate transaminase – AST; alanine transaminase – ALT; alkaline phosphatase – ALP), Immunoglobulin G, and hormone changes during pregnancy in healthy and AIH pregnant women. These changes are correlated with T helper (Th1/Th2/Th17) regulatory T cells (Tregs) immune cell subsets during pregnancy in AIH women (right figure).

Sickness ameliorates during the second trimester as the inflamed environment disseminates. This period accounts for the growth and development of the fetus. Toward the end of the third trimester when the fetus has fully developed, parturition is stimulated by the induction of a pro-inflammatory state (22). Prolactin levels increase in late pregnancy to stimulate breast milk production. The increase in islet mass is an adaptive mechanism that occurs to combat insulin resistance during pregnancy and prolactin has the ability to enhance beta-cell proliferation and insulin secretion. The prolactin receptor is required for normal glucose homeostasis and modulation of beta-cell mass during pregnancy (23). Maternal leukocytes gather in reproductive tissues and release pro-inflammatory cytokines to stimulate uterine contractions and subsequent birth of the fetus (22). Estrogen, P4, prolactin and hCG levels do not differ between healthy and AIH pregnancies. IgG, an immunological marker of AIH, increases more rapidly post-partum in AIH women as it contributes to the disease flare. ALT and AST levels do not change in healthy women but rise post-partum in a cohort of AIH women who develop a flare. ALP gradually increases throughout pregnancy in AIH women as it is produced by the placenta (Figure 1).

## Remission of AIH During Pregnancy and Immune Cells Dynamics

Pregnant women are considered to be a unique population as pregnancy creates a distinctive immunological state. The change in immune cell frequency and cytokine profile during pregnancy is related to the change in hormonal environment. In other autoimmune diseases such as multiple sclerosis and rheumatoid arthritis, disease severity decreases during gestation but increases after birth. Schramn et al. reported that 73% of 22 AIH patients experience a biochemical remission at conception (24). This is thought to be due to pregnancy hormones stimulating Th2 cells which halts the Th1 immune response linked to autoimmune disease (25, 26). A bias of T cells balance leaning toward Th2 phenotype is thought to be critical for normal pregnancy (27). The specific interaction between the sex hormones and Th2 cells requires deeper focus of exploration in the laboratory to determine the exact mechanism of pregnancy-linked autoimmune disease remission. This mechanism could also explain disease remission in pregnant AIH patients.

**FIGURE 2:**
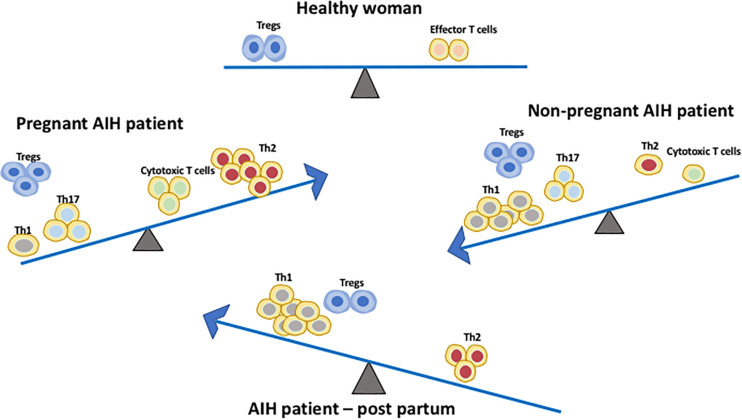
Immune cell balance in the peripheral blood differs between healthy patients, non-pregnant AIH patients, AIH patients during pregnancy and AIH patients during early post-partum. The Th1/Th17:Th2 balance shifts from Th1/Th17 predominance in AIH patients without pregnancy to Th2 predominance during pregnancy. This immune balance is switched back to Th1 predominance post-partum. Treg frequency is increased in both non-pregnant and pregnant AIH patients. However, during post-partum their frequency is comparable to normal women.

Aghaeepoor et al. utilized mass cytometry to profile both innate and adaptive immune cells in whole blood samples of 18 pregnant women collected at different trimesters throughout pregnancy and 6 weeks post-partum. There was an enrichment of circulating neutrophils, enhanced response of NK cells and monocytes to viral challenges, and a dampened TLR4 response to LPS in mDC in the innate compartment (28). It is also noted in the study that there is an abundance of CD56^+^CD16^–^ NK cells during the first trimester. NK cells are important for normal placental development and tolerance. The study demonstrated progressive increase in endogenous STAT5ab signaling across multiple T cell subsets including naïve, memory CD4 and CD8 T cells, and Treg (28). Treg are crucial in maintaining tolerance to the fetus during pregnancy. A study using pregnant mice demonstrated that depletion of Treg via the IL-2 receptor results in early resorption of allogeneic fetus (29). IL-2-dependent STAT5 signaling is also crucial for the development and function of CD25^+^Foxp3^+^ Tregs (30) which operates via the CTLA-4 pathway (31). This finding suggests that a progressive increase in endogenous STAT5 signaling activity and circulating level of Tregs play a parallel role to maintain feto-maternal tolerance during pregnancy (32). In addition, a recent study on 33 longitudinally sampled bloods in pregnancy demonstrated transient T cell polarization from Th1/Th17 to Th2, not only in the CD4 subset but also in CXCR5+ T follicular helper cells and CD8 T cells subsets (33) (Figure 2). This could potentially explain why AIH patients often experience remission during pregnancy. The suppressive capacity of CD4 + CD25^*bright*^ Treg cells was increased in the decidua of HLA-C mismatched pregnant patients. Thus, HLA genotype of the mother and fetus could also have an impact and this increase in Tregs could explain AIH remission during pregnancy. The complex network of immune cells at the decidual maternal interface and at the human decidua suggested that the activated fraction of CD8 cells are elevated but the naïve fraction are diminished. There is also a diverse DC population (34).

Th2 type cytokines, including CCL22, produced by trophoblasts, attract CCR4-expressing T cells in decidua and play a central role in the induction and maintenance of allograft tolerance during pregnancy (35). Thus Th2 cells are present predominantly in the decidua in early pregnancy in humans and its cytokines IL4 and IL6 induce the release of hCG from trophoblasts which in turn stimulate production of P4 from the corpus luteum in pregnancy (36). Th1 cells (especially Th1-dependent effector mechanisms) play a central role in acute allograft rejection in pregnancy (37).

Hormones play a major role in pregnancy and contribute to the endocrine-pregnancy-immune clock axis. During pregnancy, there is a shift from Th1 toward Th2 predominance due to a high level of circulating estrogen which explains the improved AIH disease course during gestation (38) (Figure 2). Experiments looking at the effects of pregnant mice treated with estrogen concentrations during pregnancy show that estrogen up-regulates the expression of CD4^+^CD25^+^Treg and Foxp3mRNA, thus disrupting the balance between Th1 and Th2 resulting in an enhancement of the tolerogenic immune system (39). P4 dampens immune responses to fetal and maternal antigens, and its regulatory role on Treg cells during human pregnancy was initially reported in late 2000 (40). Both *in vivo* and *in vitro* models suggest that P4 increases the proportion of CD4^+^CD25^+^Treg cells whilst simultaneously enhancing their suppressive capacity, suggesting that P4 may play a role in promoting AIH disease remission during pregnancy (41). Piccinni et al. demonstrated that P4 favors the development of Th2 CD4^+^ T cells, suggesting that P4 contributes as a factor for the Th2 predominance during pregnancy (42). Additionally, P4 and glucocorticoids inhibit Th1 development while enhancing Th2 polarity (16). P4 and testosterones have known abilities to promote Th2 cells and have anti-inflammatory properties (43). Thus, AIH remission during pregnancy may be correlated to the differential variations in complex hormonal profiles and the subsequent varying effect on immune tolerance between individuals.

Pregnant AIH patients are in an immune tolerant state. In addition, patients with AIH are already on immunosuppressive medications including steroid, azathioprine and mycophenolate mofetil (1). Enhanced immune tolerance during pregnancy sometimes may lead to susceptibility to pathogens thus these patients are prone to acquire viral infections including hepatitis E infection (44). Pregnant AIH patients who are cirrhotic may also be prone to spontaneous bacterial peritonitis. During the current COVID pandemic, the United Kingdom government and Public Health England classified both AIH and pregnancy patients as vulnerable groups and these patients were shielded^1^.

## Twin Pregnancy and Immune Response

Autoimmune hepatitis is in remission both biochemically and immunologically during pregnancy due to the Th1:Th2 balance shifts toward Th2 predominance. Th2 immunity is more pronounced in twin pregnancies compared with singleton pregnancies during the first trimester which is associated with increased maternal hCG and P4 levels (45). The data on twin pregnancies in patients with AIH are lacking therefore we can only hypothesize based on the data published to date that a highly expressed Th2 predominance in twin pregnancy will lead to a greater remission of AIH disease. During the post-partum period, the Th1:Th2 balance shifts back toward Th1 predominance. It can also be hypothesized that AIH patients who have experienced a twin pregnancy may also experience a greater shift from Th2 to Th1 predominance and thus resulting in a greater post-partum flare compared to patients who undergo a singleton pregnancy. It is likely that the mechanism of AIH pregnancy and the post-partum period may differ between twin and singleton pregnancies. Future research in this area should focus on understanding the effects of twin pregnancies in AIH patients, with particular interest in comparing remission during pregnancy and post-partum to singleton pregnancies in AIH patients. However, it is understandable that this research is limited due to the rarity of an AIH patient pregnant with twins.

## Multiple Pregnancies and AIH

The antigen-specificity and cellular origin of maternal Tregs that accumulate during gestation remain undefined. Memory T cells which recognize the antigen will play a significant role. Pregnancy selectively stimulates the accumulation of maternal Foxp3+ CD4 cells with fetal-specificity. Thus, after delivery, fetal-specific Tregs persist at elevated levels to maintain tolerance to pre-existing fetal antigen and rapidly re-accumulate during a subsequent pregnancy. The accelerated expansion of Tregs during secondary pregnancy is driven almost exclusively by proliferation of fetal-specific Foxp3+ cells retained from a prior pregnancy. Therefore, pregnancy can imprint a memory phenotype in Treg and these Treg can sustain an anergic memory response to fetal antigens in subsequent pregnancies (46). In addition, pregnancy has both short and long-term effects on the maternal memory T-cell population. Proportions of effector memory T-cells (CD45RO−CCR7+) were significantly higher in both pregnant and post-partum women compared to non-pregnant women who had never been pregnant. Pregnant women also had higher levels of activated memory T-cells, with the non-pregnant women having the lowest proportions. This suggests that the continuous exposure to fetal antigens during pregnancy results in a much higher circulating population of memory T-cells which persist after delivery (47). If the second pregnancy follows the same pattern of AIH remission during pregnancy followed by a post-partum flare, the reasoning may be that circulating memory T cells mount the same response similar to first pregnancy. It would be of interest to compare women who have had multiple pregnancies and to follow them for longer than 6 months post-partum.

## Immune Cells in the Placenta: Maternal-Fetal Interface

**FIGURE 3:**
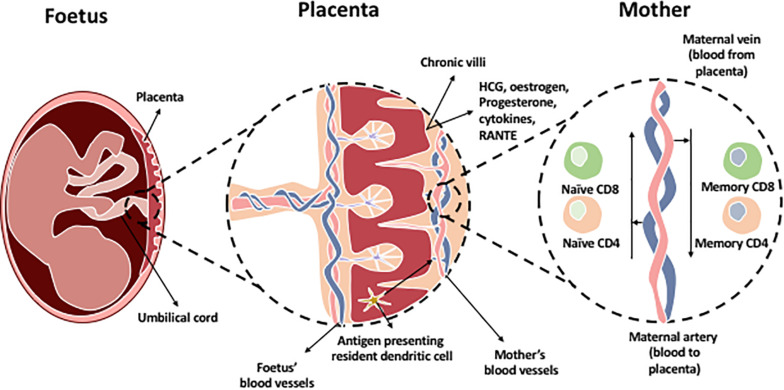
The chronic villi in the placenta contains a blood supply rich with fetal antigens additional to HCG, estrogen, progesterone hormones, and immune cells which are present in the placenta. Most predominant cells in decidual tissue is NK cells. Regulatory T cells are present to maintain tolerance at the maternal-fetal interface. In addition, antigen presenting resident dendritic cells and macrophages are found within the chronic villi which present fetal antigens to naïve T cells (CD8 and CD4) located within the maternal artery transporting blood to the placenta. The maternal vein transporting blood from the placenta contains memory CD4 and CD8 T cells as they have been exposed to the fetal antigens.

Pregnancy is characterized by the coexistence of two genetically distinct persons. The placenta is an immunological barrier between the mother and the semi-allograft; the fetus. Immune cells interact at the feto-maternal interface composed of the maternally derived decidua and the fetally derived placenta. This is crucial for maintaining tolerance of the allogenic fetus by the mother’s immune system during pregnancy. After implantation of the blastocyst, trophoblast cells burrow into the maternal decidua, which originate from the uterine endometrium. Trophoblasts secrete hCG which is required to induce progesterone from the corpus luterum. Several mechanisms involving different immune cells and molecules are involved in order to maintain placental function and survival of the fetus (Figure 3). Most of the interactions are intended to suppress the immune response and promote tolerance by preventing the miscarriage or killing of allogeneic fetus.

The most abundant immune cells present in the decidua are uterine NK (uNK) cells which are recruited by factors released from the decidual stromal cells and placental trophoblasts. The role of uNK cells is associated with decidual modeling and promote trophoblast invasion. Their cytotoxicity is controlled by the binding of HLA-G on the extravillous trophoblast to the inhibitory receptor KIR2DL4. Decidual NK cells are phenotypically different from peripheral NK cells counterpart by producing growth factors, angiogenic factors, and cytokines (48).

In addition, primary antigen presenting cells at the maternal-fetal interface in early pregnancy are decidual macrophages. They have a regulatory M2 phenotype (49) and express IL-10 and also secret indoleamine 2,3-dioxygenase which catabolizes tryptophan, subsequently preventing T cell activation (50).

Regulatory T cells are crucial in pregnancy and there is a strong inverse correlation of their frequency and function with adverse pregnancy complications such as miscarriages and spontaneous abortions (51–53). Treg are present in the decidua and modulate the activities of both antigen presenting cells and effector T cells. Fetal-specific Tregs maintain the maternal-fetal interface tolerance and their expansion correlates with decreased fetal resorption in mice models, showing Treg plays a role in tolerance (46). Regarding effector T cells, HLA-C is expressed by trophoblasts in the decidua and incompatibility between the maternal and fetal HLA-C can also alter T-cell activation during pregnancy. CD4 + CD25^*dim*^ effector T-cell frequency is increased and the percentage of these cells is increased even more in the presence of an HLA-C mismatch (54).

## Flare Up of Autoimmune Hepatitis During Pregnancy, Post-Partum and Its Underlying Immune Mechanism

A flare is defined as a two-fold increase in serum transaminase levels above the normal limit alongside elevated immunoglobulin G (13). Around 20% of AIH patients experience a flare during their pregnancy (24). Due to the return of normal immunity, AIH flares are twice as frequent in the post-partum period than during pregnancy (55). However, the underling immune mechanism for a post-partum flare still remains elusive. AIH flares during pregnancy are associated with a high rate of fetal and maternal complications (55, 56).

The current understanding of this phenomenon evolves around data concluding that flare up of AIH results from the loss of the pregnancy-associated immunosuppressive state (29, 57). Changes in the maternal immune cell subsets such as T, B, and NK lymphocytes occur during and after pregnancy. Helper T cells and cytotoxic T cells are the first cell subsets to increase 1–4 months post-partum (58). At around 7–10 months post-partum, there is an increase in frequency of the suppressor T cell and B cell populations (58). The increase in T and B cells are thought to be related to the post-partum onset or aggravation of AIH (12, 58). Schramn et al. reported that the majority of AIH flares occur at a median gestational age of 3 months after delivery (24) thus supporting the theory that an increase in effector T cells, specifically helper T cells and cytotoxic T cells, may be the main cell subsets responsible for post-partum flares. An increase in suppressor T cell and B cell populations could play a role in the recovery from a post-partum flare.

Many immune-mediated diseases are ameliorated by the process of pregnancy and are coupled with subsequent flares following parturition. As previously stated, CD4^+^CD25^+^Treg are a subset of CD4 T cells with immunosuppressive and tolerogenic functions (59, 60). An early report by Aluvihare et al. on Treg suggested that they regulate maternal immune tolerance to the fetus; which is an allo-antigen to the mother’s immune system (29). Thus, Treg cells are necessary to maintain maternal-fetal immune tolerance to ensure that the pregnancy is successful. A number of studies have since confirmed that Treg frequency is increased in the maternal peripheral blood and play a crucial role in the maternal-fetal immune tolerance (36, 61). In addition, proportion of CD4^+^CD25^+^Treg in pregnant women is significantly higher compared to non-pregnant women but decreased significantly after birth compared to non-pregnant women (62). Estrogen also mediates Treg cell expansion via Estrogen receptor alpha (ERα) signaling and the transcriptional regulation of Foxp3 which subsequently induces Tregs suppressive function (39). This provides a potentially strong explanation for remission of AIH patients during pregnancy, often followed by a post-partum disease flare. The shift of immune balance to Th1 predominance is noted after a decline in blood concentration of estrogen following delivery, and this could be an additional reason for a flare in AIH (63) (Figure 2). Although the effect of high level estrogen during pregnancy was thought to induce tolerance, more recent investigations have shown that heightened levels of estrogen receptor alpha (ERα) is leading to the impairment of Tregs in AIH (64). A fall in P4 also leads to lack of inhibition of Th1 development which could result in a post-partum flare in AIH patients (16). The presence of fetal cells, which remain in maternal circulation for years after birth, may play a role although the specific mechanism and extent of their presence in relation to AIH flares post-partum is still unclear (25).

There is a correlation between women with AIH who experience a flare in the year before conception and those who experience post-partum flares (10). If we were able to identify the similarities in cell subsets between a flare before pregnancy and a post-partum flare, we may be able to predict those patients who are at high risk to post-partum flares before it occurs. This will provide us with sufficient time to monitor and adjust immunosuppression which will likely prevent the post-partum flare and subsequent liver fibrosis. Women with AIH who are not currently undergoing immunosuppressive therapy are also at a higher risk for experiencing a flare post-partum (10).

## Current Proposed Mechanisms of Tolerance and Immune Escape

**FIGURE 4:**
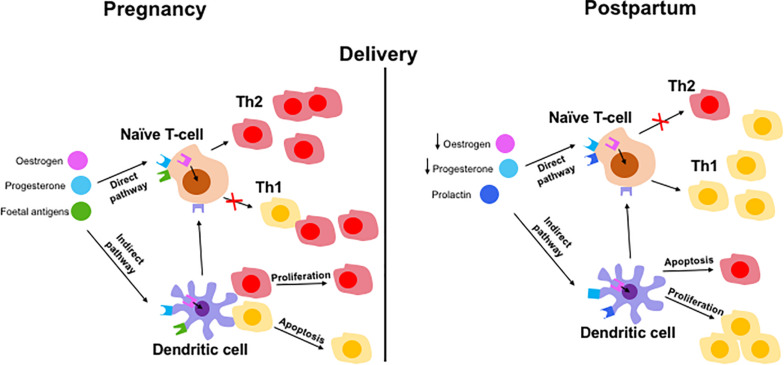
Hypothetical mechanisms of immune tolerance in pregnancy and immunological escape at post-partum in AIH patient. Maintaining tolerance to the fetus by the mother via adaptive immune cells involving both direct and indirect pathways. Direct pathways include pregnancy hormones and fetal antigens presenting to naïve T cells which results in a Th2 predominance phenotype during pregnancy. Lack of hormones and fetus results in switching back to Th1 dominant immune balance. Indirect pathway involve hormones and fetal antigen priming to naïve cells via antigen presenting dendritic cells resulting in Th2 cells proliferation and Th1 cells apoptosis (in pregnancy) and reverse effect during post-partum period.

Currently, there is only limited research in the field of immune tolerance in pregnant AIH patients. The immunome changes should be longitudinally observed throughout pregnancy and post-partum period. So far, the evidence suggests that rising levels of estrogen increase the expression of CD4+ CD25+ Tregs which shifts the immune balance to favor Th2 cells (25, 39). This is further supported by an increase in circulating IL-2 in pregnant women which increases Treg proliferation via STAT5 signaling (28). The suppressive capacity of Tregs has also been shown to be increased in pregnant patients (54). These changes are important for maintaining feto-maternal tolerance (61) but are also most likely responsible for the amelioration of AIH symptoms in pregnant patients. It is possible that naïve T-cells differentiate into Th2 cells after stimulation by estrogen, P4 and fetal antigens (Figure 4). These factors can also stimulate DCs which in turn stimulate naïve T-cells. Fetal antigen receptors are extracellular receptors whereas P4 and estrogen receptors are intracellular. After delivery, the fall in estrogen and P4 and the presence of prolactin may exert Th1 prominence via naïve T-cells or DCs (Figure 4).

## Associated Autoimmune Diseases and Pregnancy

Rheumatoid arthritis, multiple sclerosis and psoriasis are both considered to be Th1− and Th17−dominant diseases, thus a decline in Th1 and possibly Th17 during pregnancy is expected to lead to the improvement of these diseases similar to AIH. Improved outcomes in patients with rheumatoid arthritis (65), multiple sclerosis (66), and psoriasis (67) was observed and a relapse after delivery has been reported possibly due to the reconstitution of Th1/Th17 immunity post−partum. Normal human pregnancy is associated with an elevation in the immune suppressive CD25+ CD4+ regulatory T−cell subset, thus this relapse could occur because of the reduced levels of Tregs (61).

On the other hand, autoimmune diseases such as asthma, actopic dermatitis, systemic lupus erythematosus (SLE), and pemphigus are Th2− and/or Th17−dominant autoimmune diseases (68–71). These patients experience the exacerbation of disease during pregnancy. Although Th17 is a known player in these autoimmune diseases, these diseases flared up during pregnancy. This may be due to the higher importance of Th2 compared with Th17. It could also be explained with the possible increased populations of Th17 cells in pregnancy, which is still under investigation. Systemic lupus erythematosus generally affect women of reproductive age. Autoimmune hepatitis was initially known as lupoid hepatitis due to it’s association of chronic active hepatitis in SLE patients. Some of the AIH patients have a coexisting SLE condition. A previous study suggested diminished CD4^+^ Tregs were observed in pregnant lupus patients which might be another important factor responsible for pregnancy associated complications. Although AIH patients experience disease remission during pregnancy, other autoimmune diseases may have a flare up due to underlying immune cell balance changes. This aspect of mutiorgan autoimmune disease flare up or remission will require further research.

## Estrogen and Its Signaling in Pregnancy

Molecular and metabolic immunological changes that occur during pregnancy are influenced by a variety of hormones which leads to intracellular signaling and results in pregnancy induced tolerance. Estrogen bind to two nuclear estrogen receptors (ERs) (ERα and ERβ)which are expressed in T cells and activate intracellular signaling pathways. Estrogen has a different impact on T cells. Pregnancy-level concentrations of estrogen restrain the conventional Foxp3^*neg*^ CD4^+^ T cells to differentiate to Th17 cells (72). ERα signaling in T cells is necessary for E2-mediated inhibition of Th1/Th17 cell differentiation and protection from neurological autoimmune diseases in mice (72). This may explain the reason of improvement in MS in pregnancy. ERα can contribute to T cell-mediated autoimmune inflammation by promoting T cell activation and proliferation (73) thus ERα-targeted immunotherapies could be used to treat autoimmune disorders.

## Role of Th17 Cells in Pregnancy

Autoimmune diseases such as rheumatoid arthritis, inflammatory bowel disease, multiple sclerosis and autoimmune skin diseases are not only solely Th1 driven diseases; Th17 cells have also been implicated in these disorders. There is evidence indicating that sex hormones can influence Th17 signaling. Multiple sclerosis (MS), a T cell–mediated autoimmune disease, is such an example. Reduced disease activity of MS is commonly observed during pregnancy, suggesting that estrogens could downmodulate the autoimmune response and inflammation (74–76). Protective effects of estrogens have been reported in clinical trials using estriol or 17β-estradiol (E2) (77–80) in relapsing-remitting multiple sclerosis patients. The growing knowledge suggests that AIH is multi-facetted immune dysregulation, which involves Th1/Th17 polarization and the suspected inability of regulatory T cells to revert autoimmunity. Manipulation of the Th1 axis with standard-of-care immunosuppressive medications and also blocking IL17 and IL22 may be important in preventing post-partum flare up in AIH patients in the future.

## Conclusion and Future Perspectives

Women with autoimmune hepatitis who wish to become pregnant must be closely monitored by an interprofessional team including hepatologists due to the increased risk of adverse outcomes. Pregnancy is becoming more common in AIH patients as the majority of the cohort are young women. It is now accepted that there is a decreased risk in AIH owing to close surveillance and clinical, medical and technological improvements associated with positive outcomes for both mother and child. To further decrease the accompanying risks, it would be beneficial to broaden our understanding of the immunology aspects involved with remission, flares and recovery from flare up. This will not only provide us with a reliable immunological biomarker, giving clinicians guidance to predict, diagnose and treat adverse effects including flare up of AIH, but it may also enable the team to manipulate cell subsets with immune based therapy to avoid adverse outcomes alongside the continued support of an interprofessional team.

Current diagnostic markers for AIH include biochemistry, liver histology and immunology (IgG and autoantibodies) as suggested by International Autoimmune Hepatitis Group (IAIHG). However, it is still difficult to predict which patient group will experience a flare up during pregnancy and post-partum. Thus, new predictive markers in both peripheral blood compartment and tissue compartment (liver) are crucial. Recent advances in OMICs (genomics, transcriptomics, proteomics, and metabolomics) and deep immunophenotyping technology could be utilized to predict the outcome and prognosis of the clinical pathway of these patients. Mass cytometry allows the identification of a wide variety of both intracellular and extracellular markers hence making it the ideal method to determine the differences in immune cell expression in these samples. Currently it is not possible to predict a flare of AIH on those who are already on immunosuppression. In the future, this technology may pave the way to predict the flare precisely and tailor the adjustment of immunosuppression. In addition, immunological analysis of blood samples from healthy pregnant, healthy non-pregnant and AIH non-pregnant samples at different stages throughout pregnancy may also predict who could develop post-partum flare by dissecting the immunome.

## Author Contributions

GW and AB contributed equally to the content of the review and figures. OP contributed to the content of the review. YO supervised, edited, and referenced the review. All authors contributed to the article and approved the submitted version.

## Conflict of Interest

The authors declare that the research was conducted in the absence of any commercial or financial relationships that could be construed as a potential conflict of interest.

## Footnotes

1 https://www.gov.uk/government/publications/guidance-on-shielding-and-protecting-extremely-vulnerable-persons-from-covid-19/guidance-on-shielding-and-protecting-extremely-vulnerable-persons-from-covid-19
